# Improving surgical quality of care: learning from 8,331 surgical medical malpractice cases

**DOI:** 10.3389/fmed.2024.1486451

**Published:** 2024-12-10

**Authors:** Qin Chen, Xiaoyu Liu, Xiaoyan Liu, Pan Song, Xiaoyan Quan, Huarong Xiong, Dan Wang, Xiaoli Hu, Hua Zhang, Meihong Shi

**Affiliations:** ^1^Department of Spine Surgery, The Affiliated Hospital of Southwest Medical University, Luzhou, China; ^2^School of Nursing, Southwest Medical University, Luzhou, China; ^3^Department of Endocrinology, The Affiliated Hospital of Southwest Medical University, Luzhou, China; ^4^Department of Respiratory and Critical Care Medicine, The Affiliated Hospital of Southwest Medical University, Luzhou, China; ^5^Department of Gastroenterology, The Affiliated Hospital of Southwest Medical University, Luzhou, China; ^6^Law School,Southwest Medical University, Luzhou, China; ^7^Department of Nursing, The Affiliated Hospital, Southwest Medical University, Luzhou, China

**Keywords:** medical malpractice, medical damage liability disputes, compensation, surgery, China

## Abstract

**Objective:**

This study aimed to analyze the characteristics of surgical litigation cases and the risk factors that contribute to catastrophic compensation.

**Methods:**

We downloaded and retrieved all cases related to surgical litigation cases from the China Jufaanli Database between 2008 and 2023. Multivariate logistic regression analysis was employed to identify independent risk factors that may contribute to catastrophic compensation.

**Results:**

This study included a total of 8,331 successfully resolved surgical litigation cases. Of these, 5,114 hospitals were defendants, with 25.34% of them involved in two or more lawsuits, thereby categorized as “repeat defendants.” The total compensation amount was $269,163,545, with the highest compensation reaching $540,008. Most surgical litigation cases were concentrated in the eastern regions of China, with tertiary hospitals being the most frequently involved. The most common type of injury outcome was patient death. Compensation amounts and high compensation rates for severe disability exceeded those for death (*p < 0.05*). Independent risk factors associated with catastrophic compensation in surgical medical liability disputes included: Eastern region (OR = 1.462, 95% CI 1.038–2.060), secondary liability (OR = 2.457, 95% CI 1.633–3.696), main liability (OR = 9.353, 95% CI 6.195–14.121), major or full liability (OR = 10.878, 95% CI 7.152–16.546), severe disability (OR = 24.605, 95% CI 3.395–178.337), neurosurgery (OR = 3.488, 95% CI 2.265–5.373), thoracic surgery (OR = 1.810, 95% CI 1.017–3.219), general surgery (OR = 2.465, 95% CI 1.593–3.816), hepatobiliary surgery (OR = 3.251, 95% CI 1.980–5.338), gastrointestinal surgery (OR = 2.260, 95% CI 1.391–3.671), cardiovascular surgery (OR = 2.544, 95% CI 1.367–4.733), vascular surgery (OR = 2.916, 95% CI 1.246–6.827), and spinal surgery (OR = 2.921, 95% CI 1.763–4.841).

**Conclusion:**

This study analyzes the characteristics of surgical medical malpractice disputes in China from multiple perspectives and identifies independent risk factors for catastrophic compensation in surgical malpractice litigation. Our research has the potential to aid medical institutions in preventing and reducing surgical malpractice disputes, while also contributing to the provision of improved surgical care and nursing services for patients.

## Introduction

Medical disputes have long been a significant concern globally and are an urgent issue requiring resolution (1–3). Among medical specialties, surgery has emerged as the highest-risk area for medical liability disputes (4, 5). In mainland China, surgical medical liability disputes account for 45.4% of all medical dispute cases (6). Similarly, in other regions and countries, surgical medical liability disputes represent the largest proportion of medical dispute litigation cases. In Taiwan, the incidence of surgical medical disputes is 39.4% (7). A retrospective study conducted in Germany found this proportion to be as high as 66.5% (8). Additionally, research from other European countries confirms that surgery is the specialty with the highest incidence of medical disputes (9, 10). Surgery, characterized by invasive procedures, critical conditions, complex cases, and a high rate of complications, inherently carries a higher risk compared to other specialties. This high-risk nature makes surgery a frequent source of medical disputes, which can adversely affect medical institutions and healthcare professionals when such disputes arise.

The high incidence of surgical medical dispute litigation globally imposes significant professional stress on surgeons. A study involving 7,000 surgeons in the United States found that medical dispute litigation profoundly impacts physicians, leading to burnout, career changes, and even suicide (11). Research in Japan also indicates that the high frequency of surgical medical disputes similarly puts considerable pressure on Japanese surgeons, resulting in a decrease in the number of practicing surgeons and a 21% attrition rate (12). Moreover, the high occurrence of surgical medical disputes severely affects doctor-patient relationships, making surgery the specialty with the highest proportion of defensive medicine practices (13). In a study on defensive medicine in the United States, approximately 93% of physicians engaged in defensive practices (14). It is estimated that defensive medicine incurs an additional cost of $46 billion annually in the U.S. (15). Unfortunately, research on defensive medicine in China is limited, and there are no comprehensive data on its impact on healthcare expenditures in China. However, it is known that over 80% of doctors in China report employing defensive medicine measures to avoid being defendants in medical disputes (16). Defensive medicine refers to the practice of ordering tests, procedures, or referrals, or avoiding high-risk patients or surgeries primarily to prevent litigation rather than for medical purposes (17). This practice has negative effects on patients, doctors, and the healthcare system, including increased hospital stays, higher care costs, damage to doctor-patient relationships, and unnecessary use of scarce medical resources.

Although numerous studies have revealed the characteristics of surgical medical dispute litigation across various countries, several limitations remain. For instance, some studies focus solely on specific surgical departments (18–22), others on particular diseases (23–27), and some are restricted to surgical disputes within a single region (28, 29). Moreover, the number of surgical medical dispute cases examined in these studies is relatively limited (ranging from 28 to 1,550 cases) (Supplementary Table S2), which makes it difficult to comprehensively describe the overall trends and characteristics of surgical medical liability disputes. Therefore, this study aims to address these gaps by providing a multi-dimensional analysis of the features and compensation trends of surgical medical liability disputes in China, based on a large sample of 8,331 cases. This research seeks to offer valuable insights for medical institutions to better identify and prevent surgical medical liability disputes. Specifically, by analyzing existing evidence, this study aims to identify the risk factors associated with surgical malpractice disputes and catastrophic compensations, thereby facilitating the management of related risks to mitigate the substantial economic burden posed by surgical malpractice disputes (30, 31), thereby supporting the effective progression and development of surgical healthcare services.

## Materials and methods

### Data sources

The original cases were sourced from the China Jufaanli Database,^1^ a specialized intelligent legal database and application platform. All cases related to surgical medical disputes from 2008 to 2023 were downloaded.

### Case retrieval

Two researchers (QC & XL), both with a background in surgical medical dispute research, conducted an independent systematic search using keywords such as “surgery,” “surgical,” “medical liability disputes,” “medical disputes,” “doctor-patient disputes,” and “compensation” to retrieve and download cases. Subsequently, both researchers (QC & XL) independently reviewed the titles and abstracts of all retrieved cases to exclude those that did not meet the inclusion criteria.

### Data extraction

A custom-designed Excel data extraction form was utilized. Two researchers (QC & XL) independently reviewed the full text of each remaining case and extracted the following key information: case number, province, judgment date, name of the defendant hospital, hospital level, liability degree of the hospital, surgical department, injury outcome, and compensation amount.

### Definition and coding of variables

This study includes medical malpractice liability disputes from 30 provincial administrative units across China. According to China’s economic regional classification, these provinces and municipalities are categorized into Eastern, Central, Western, and Northeastern regions. The Eastern region is the earliest to adopt open-door policies and has the highest level of economic development. The Central region represents the second most economically developed areas, while the Western region is characterized by relatively lower economic development. The Northeastern region comprises only Liaoning, Shenyang, and Heilongjiang provinces.

Hospital level is based on the “Hospital Grading Management Standards” in China, which assesses hospitals’ qualifications according to their scale, personnel, medical hardware and equipment, research and medical technology levels. Hospitals are classified into four levels: (1) Private hospitals, typically operated by individuals or private institutions with a commercial purpose; (2) Primary hospitals, which are community-based facilities providing primary health care, with a total number of inpatient beds ranging from 20 to 99; (3) Secondary hospitals, which serve as regional institutions beyond community boundaries, with a total number of inpatient beds ranging from 100 to 499; and (4) Tertiary hospitals, which operate across provincial or municipal boundaries and have more than 500 inpatient beds. Primary, secondary, and tertiary hospitals are all government-funded and operated, with the overall strength of these institutions increasing with each level.

“Liability degree of the hospital” refers to the proportion of liability attributed to the defendant hospital in medical malpractice cases (1–100%). The higher the hospital’s degree of liability, the more significant error in the malpractice. “Liability degree of the hospital” is broken down into the following 4 categories: minor liability (≤25% or less), secondary liability (25–50%), main liability (51–75%), major or full liability (76–100%).

“Injury outcomes” refer to the detailed consequences of medical accidents. In this study, the injury levels are categorized into five classes, which classify the severity of physical harm to patients. The ten levels of disability and other outcomes are consolidated into the following five categories to measure “injury”: “Minor disability” (levels 10–7, with partial loss of self-care ability), “Moderate disability” (levels 6–5, with significant loss of self-care ability), “Severe disability” (levels 4–1, with complete loss of self-care ability), “Death,” and “Serious illness”(Changes in patient condition without resulting in disability) [According to Medical Accident Classification Criteria (Trial) (32)].

In this study, hospitals that have been defendants in two or more surgical malpractice cases are defined as repeat defendants. Cases involving compensation amounts exceeding $100,000 are classified as catastrophic compensation cases, also referred to as high compensation cases (33).

### Statistical analysis

Data collection and organization were performed using Excel 2016, while OriginPro 2024 was employed for graphical analysis. Statistical analysis was conducted using SPSS 27.0. The compensation amounts, being continuous data, were tested for normality. As the data did not follow a normal distribution, median and interquartile range were used for statistical description. Other categorical variables were described using frequency and percentage frequency. Differences between groups were assessed using the χ^2^ test and *Fisher’s* exact test. Factors potentially leading to catastrophic compensation in surgical malpractice cases were analyzed using multivariate logistic regression, with results presented as odds ratios (OR) and 95% confidence intervals (*CI*). A *p*-value of less than 0.05 was considered statistically significant.

## Results

**Figure 1:**
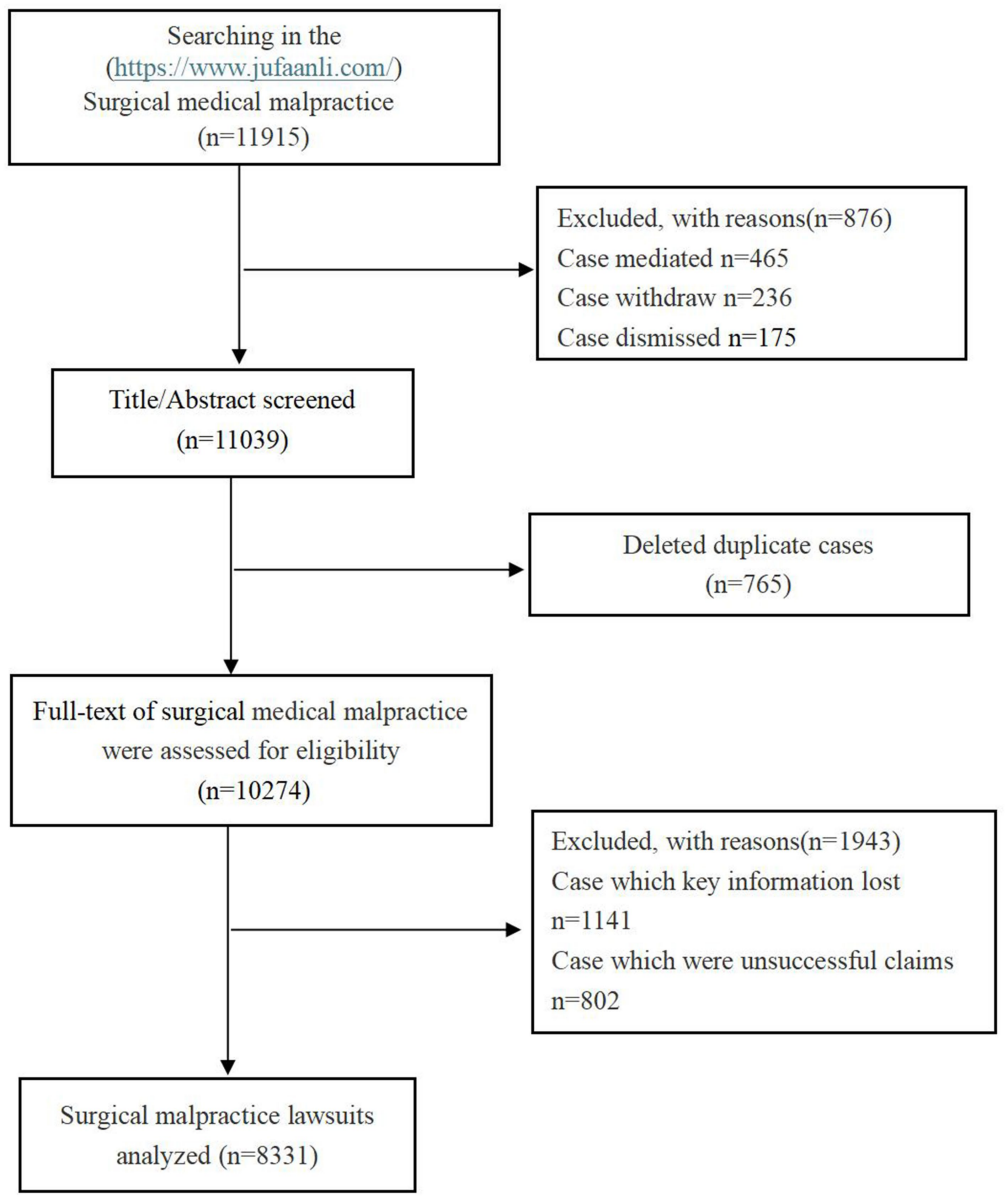
The flow diagram for the inclusion of surgical malpractice lawsuits.

A total of 11,915 cases of surgical medical litigation were identified. After reviewing the titles and abstracts, 876 cases resolved through mediation, withdrawn, or dismissed were excluded. After reviewing the titles and abstracts, 765 duplicate cases were removed. Following a thorough review of the full texts, 1,141 cases with missing key information and 802 cases with unsuccessful compensation were excluded. Ultimately, 8,331 surgical medical malpractice cases were included for analysis. The flowchart detailing the case selection process is shown in Figure 1.

### Characteristics of surgical medical dispute cases

#### Geographic distribution of defendant hospitals

In this study, a total of 8,331 cases of successfully claimed surgical malpractice disputes occurring from 2008 to 2023 were included, involving 5,114 hospitals in China as defendants. These hospitals are distributed across 30 provinces and municipalities (cases from Tibet, Hong Kong, Macau, and Taiwan were not retrieved). The three provinces and municipalities with the highest number of surgical dispute cases are Jiangsu (768 cases, 9.22%), Beijing (766 cases, 9.19%), and Shandong (701 cases, 8.41%). Among the 5,114 defendant hospitals, 25.34% (1,296/5,114) had been involved in two or more surgical malpractice lawsuits, referred to as “repeat defendants” (Table 1), with the hospital facing the highest number of lawsuits totaling 63. Within the group of repeat defendant hospitals, the vast majority (81.87%) had been sued five times or fewer; however, 18.13% (235/1,296) of these hospitals faced more than five lawsuits. Among the repeat defendant hospitals involved in this study, tertiary hospitals accounted for the highest proportion, constituting 70.60% (915/1,296) of all repeat defendants, followed by secondary hospitals at 25.77% (334/1,296). Private hospitals had the lowest likelihood of being repeat defendants, at only 1.62% (21/1,296).

**Table 1:** Characteristics of surgical medical dispute cases.

Characteristic	*n*/%/Median (IQR)
Cases, *n*	8,331
Provinces, *n*	30
Hospital, *n*	5,114
Repeat defendant hospitals	25.34 (1,296/5114)
Repeat with 2–5 lawsuits	81.87 (1,061/1296)
Repeat with >5 lawsuits	18.13 (235/1296)
Hospital level of repeat defendant	
Private hospitals	1.62 (21/1296)
Primary hospitals	2.01 (26/1296)
Secondary hospitals	25.77 (334/1296)
Tertiary hospitals	70.60 (915/1296)
Indemnity payment	20,252 (7931–41,514)
Maximum payment	540,008
Total indemnity payment	269,163,545

IQR, interquartile range.

#### Temporal distribution of surgical medical dispute cases

**Figure 2:**
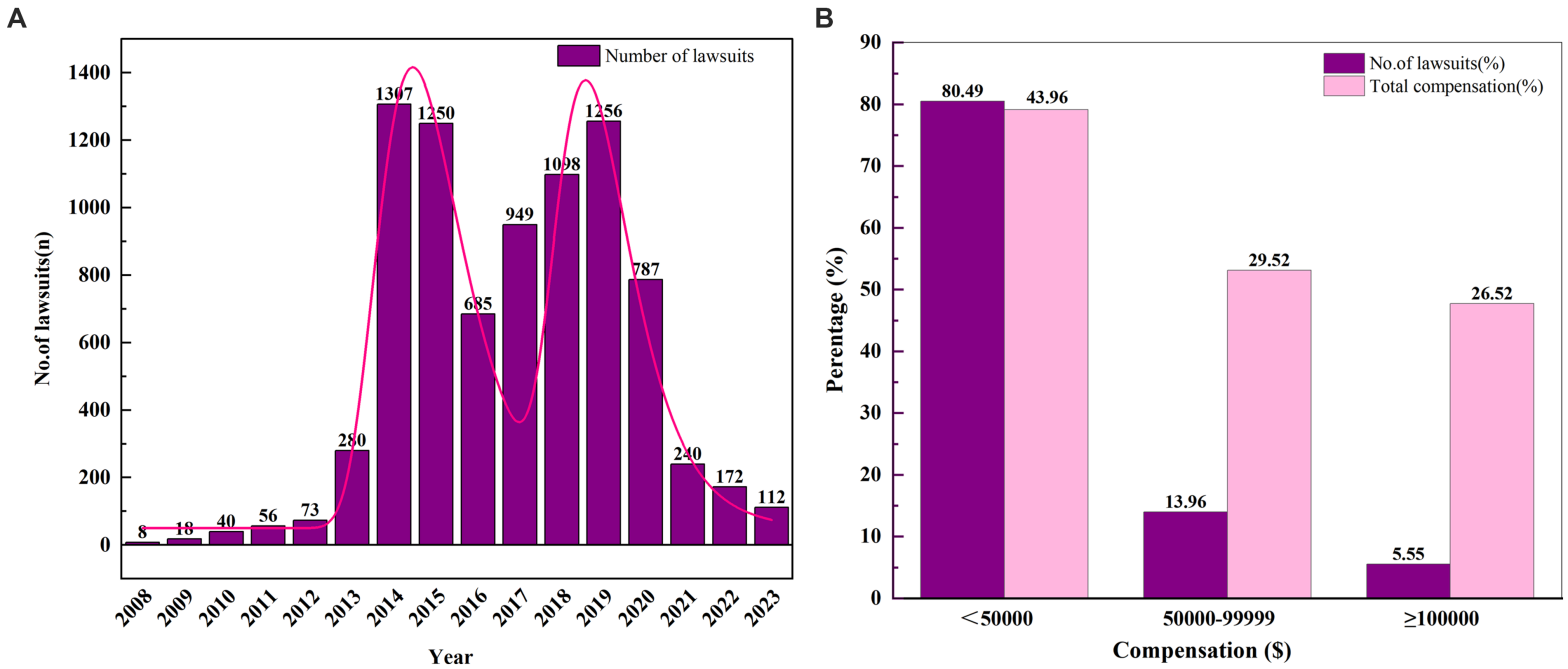
Overview of surgical medical liability dispute cases. **(A)** Surgical medical litigation cases (2008–2023); **(B)** Compensation for surgical medical disputes.

Since 2008, the number of surgical medical malpractice cases in China has shown a yearly increase (Figure 2A), reaching its first peak in 2014. Although the number of cases slightly decreased after this peak, the overall volume of surgical malpractice disputes remained substantial, with a second peak occurring in 2019, resulting in a “bimodal” distribution pattern. During the five-year period from 2014 to 2019, the number of surgical medical malpractice cases accounted for 78.56% (6,545/8,331) of the total litigation cases, indicating that this period was characterized by a high incidence of surgical medical liability disputes. However, starting from 2020, there was a sharp decline in the number of cases.

#### Compensation in surgical medical liability disputes

In this study, involving 8,331 cases of surgical medical disputes in China, the total compensation amount was $269,163,545 (Table 1), with a median compensation amount of $20,252. The highest compensation amount in a single case reached $540,008, which is approximately 26.66 times the median compensation amount. As shown in Figure 2B, the majority (80.49%) of surgical litigation cases had compensation amounts below $50,000, accounting for 43.96% of the total compensation. Cases with compensation amounts ranging from $50,000 to $100,000 constituted 13.96% of the cases, representing 29.52% of the total compensation. Although only 462 cases (5.55%) had compensation amounts exceeding $100,000, this segment of cases accounted for $71,392,976, or 26.52% of the total compensation.

### Factors influencing catastrophic compensation in surgical medical disputes

#### Injury outcomes in surgical medical disputes

Among the surgical medical dispute cases, the most common injury outcome is patient death, accounting for 56.99% (4,748/8,331) of all cases. The total compensation amount for death-related cases is $153,851,405. The second most common outcome is minor disability, representing 22.18% (1,848/8,331) of cases, with a total compensation amount of $39,704,755. In terms of catastrophic compensation, although cases resulting in severe disability account for only 11.96% (996/8,331) of the total, the compensation amount for these cases is $57,809,456, which constitutes 21.48% of the total compensation amount. The median compensation amount for severe disability cases is the highest among the outcomes, exceeding the median compensation amount for death cases (*p < 0.001*). Specifically, cases resulting in level 1 disability have the highest rate of high compensation, reaching 27.95%, with a median compensation amount nearly 2.24 times that of the median compensation for death cases. Cases resulting in s level 2 disability have a high compensation rate of 20%, with a median compensation amount 2.09 times that of the death cases. Although cases involving moderate disability account for only 4.40% of the total, the median compensation amount remains higher than that for death cases (*p < 0.001*) (Table 2).

**Table 2:** Injury outcomes and compensation in surgical medical liability disputes.

Injury outcomes	No lawsuits, *n*(%)	Without high compensation, *n*(%)	With high compensation, *n*(%)	Median (IQR) indemnity compensation, $
Death	4,748 (56.99)	4,506 (94.90)	242 (5.10)	21,343 (7,990–44,040)
Disability grade				
Severe disability	996 (11.96)	817 (82.03)	179 (17.97)	34,502 (13757–78,396)
First level disability	347 (4.17)	250 (72.05)	97 (27.95)	47,743 (19,208–105,836)
Second level disability	200 (2.40)	160 (80.00)	40 (20.00)	44,546 (18,922–85,867)
Third level disability	236 (2.83)	206 (87.29)	30 (12.71)	27,808 (11,594–65,298)
Fourth level disability	213 (2.56)	201 (94.37)	12 (5.63)	20,796 (5,714–47,302)
Moderate disability	366 (4.40)	346 (94.54)	20 (5.46)	31,331 (16355–49,895)
Fifth level disability	192 (2.30)	178 (92.71)	14 (7.29)	34,878 (19,279–54,568)
Sixth level disability	174 (2.09)	168 (96.55)	6 (3.45)	26,436 (14,165–44,026)
Minor disability	1848 (22.18)	1828 (98.92)	20 (1.08)	16,061 (8162–28,404)
Seventh level disability	348 (4.18)	337 (96.84)	11 (3.16)	25,437 (14,480–42,141)
Eighth level disability	480 (5.76)	477 (99.38)	3 (0.62)	22,375 (10,823–32,344)
Ninth level disability	529 (6.35)	528 (99.81)	1 (0.19)	14,729 (7,986–24,466)
Tenth level disability	491 (5.89)	486 (98.98)	5 (1.02)	9,324 (5,566–16,773)
Serious illness	373 (4.47)	372 (99.73)	1 (0.27)	3,673 (1,419–10,533)

*χ2 = 385.560; p < 0.001.*

### Regional distribution of surgical medical dispute litigation cases

**Figure 3:**
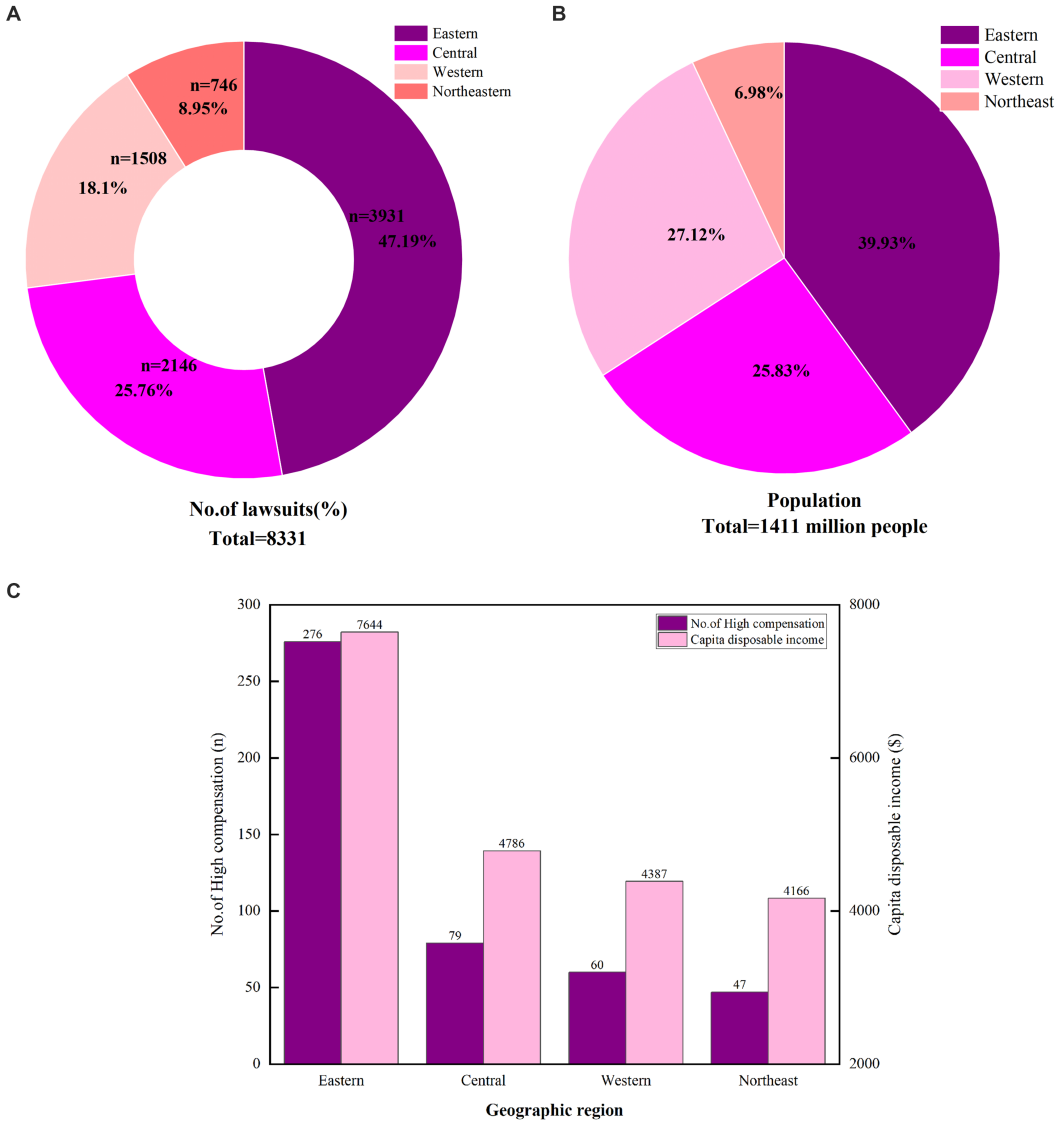
The distribution of surgical malpractice lawsuits. **(A)** The proportion of the surgical malpractice lawsuits in Eastern, Central, Western, and Northeastern Regions of China. **(B)** Population distribution in the Eastern, Central, Western, and Northeastern Regions of China. **(C)** High compensation cases and *per capita* disposable income in different regions of China.

A total of 47.19% (3,931/8,331) of the surgical medical malpractice cases occurred in the eastern region of China (Figure 3A). This is followed by the central region of China, which accounts for 25.76% (2,146/8,331) of the cases. The western and northeastern regions have fewer cases, with proportions of 18.10% (746/8,331) and 8.95% (746/8,331), respectively. This distribution aligns with the population density in China. According to the 2021 Seventh National Census of China (National Bureau of Statistics website),^2^ the population is predominantly concentrated in the eastern region, which holds 39.93% of the national population (Figure 3B).

Analysis of catastrophic compensation cases indicates that the highest proportion of such cases occurred in the eastern region of China, accounting for 59.74% (276/462) of the total (Table 3). The central region follows with 17.10% (79/462) of the cases. The western and northeastern regions account for 12.99% (60/462) and 10.17% (47/462), respectively. Notably, the eastern region is the most economically developed area in China, with a *per capita* disposable income 1.6 to 1.8 times higher than that of the other three regions (Figure 3C). (National Bureau of Statistics website: www.stats.gov.cn).

**Table 3:** Characteristics of the surgical malpractice lawsuits and the high compensation analysis.

Characteristic	Total, *n* (%)	Median (IQR) indemnity compensation, $	Without high compensation (<700,000 Yuan), *n* (%)	With high compensation (≥700,000 Yuan), *n* (%)	*χ* ^2^	*p*
Geographic region					38.458	<0.001
Eastern	3,931 (47.19)	20,821 (7,567–44,828)	3,655 (92.98)	276 (7.02)		
Central	2,146 (25.76)	19,222 (8,438–36,569)	2067 (96.32)	79 (3.68)		
Western	1,508 (18.10)	19,231 (7,846–38,452)	1,448 (96.02)	60 (3.98)		
Northeastern	746 (8.95)	21,200 (8,345–44,659)	699 (93.70)	47 (6.30)		
Hospital level					31.443	<0.001
Private hospitals	388 (4.66)	9,885 (4296–25,013)	377 (97.16)	11 (2.84)		
Primary hospitals	335 (4.02)	12,818 (4221–25,861)	326 (97.31)	9 (2.69)		
Secondary hospitals	1920 (23.05)	17,735 (7293–35,189)	1847 (96.20)	73 (3.80)		
Tertiary hospitals	5,688 (68.27)	22,677 (8994–45,954)	5,319 (93.51)	369 (6.49)		
Liability degree of the hospital					261.789	<0.001
Minor liability (≤25%)	1985 (23.83)	12,327 (5698–22,952)	1955 (98.49)	30 (1.51)		
Secondary liability (26–50%)	3,696 (44.36)	21,664 (8127–42,204)	3,564 (96.43)	132 (3.57)		
Main liability (51–75%)	1,506 (18.08)	29,097 (13134–60,308)	1,347 (89.44)	159 (10.56)		
Major or full liability (76–100%)	1,144 (13.73)	25,246 (9474–59,469)	1,003 (87.67)	141 (12.33)		
Total	8,331	20,252 (7932–41,514)	7,869 (94.45)	462 (5.55)		

#### Impact of hospital level on compensation

**Figure 4:**
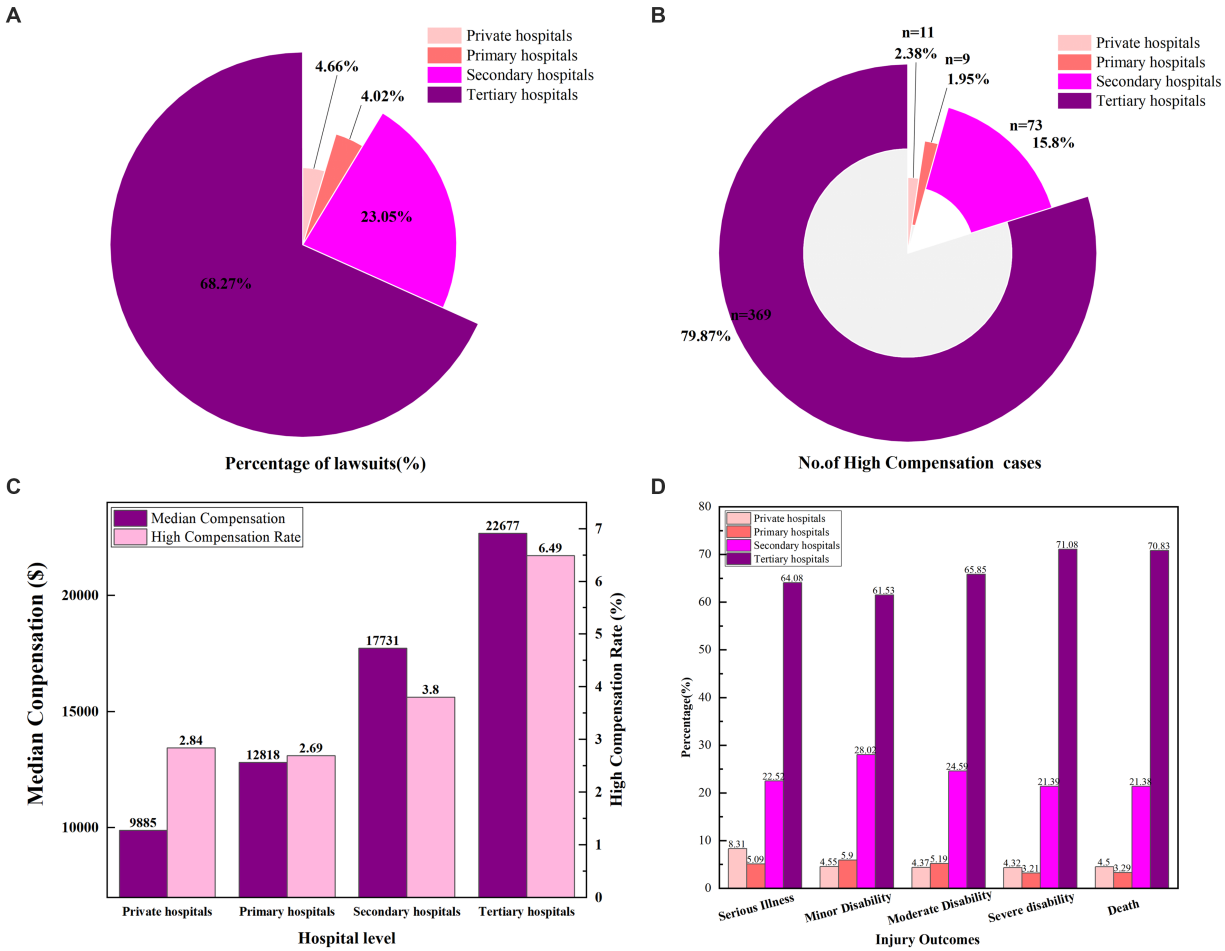
**(A)** Proportion of surgical medical disputes cases in different levels of hospitals. **(B)** Number of high compensation cases in different levels of hospitals. **(C)** Median compensation and high compensation rates for surgical cases in different hospitals. **(D)** Injury outcomes of different levels of hospital.

Surgical medical malpractice litigation cases are most frequently reported in tertiary hospitals, representing 68.27% (5,688/8,331) of all cases (Figure 4A and Table 3), with a median compensation amount of $22,677. Cases occurring in secondary hospitals account for 23.05% (1,920/8,331), with a median compensation amount of $17,735. The number of cases in primary hospitals and private hospitals is relatively low, comprising 4.02% (335/8,331) and 4.66% (388/8,331) of the total, respectively. The median compensation amount for primary hospitals is $12,818, while private hospitals have the lowest median compensation amount at $9,885. Additionally, catastrophic compensation cases are most prevalent in tertiary hospitals (Figure 4B), accounting for 79.88% (369/462) of such cases. Tertiary hospitals also have the highest rate of high compensation claims (6.49%), which is nearly twice the high compensation rate of secondary hospitals and three times that of primary and private hospitals (Figure 4C and Table 3).

The analysis of injury outcomes in surgical medical malpractice cases shows that tertiary hospitals have the highest proportions of all types of injury outcomes (Figure 4D). Specifically, tertiary hospitals account for 70.83% (3,363/4,748) of deaths; 71.08% (708/996) of severe disabilities; 65.85% (241/366) of moderate disabilities; and 61.53% (1,137/1,848) of minor disabilities and 64.08% (239/373) of cases with exacerbation of conditions. This indicates that the majority of severe injury outcomes in surgical medical malpractice cases occur in tertiary hospitals.

#### Liability degree of defendant hospitals

In this study, defendant hospitals were most frequently found to bear secondary responsibility, accounting for 44.36% (3,696/8,331) of the cases (Table 3). However, the high compensation rate in these cases was 3.57%. Despite the lower frequency of cases where hospitals bore main liability 18.08% (1,506/8,331), the median compensation amount was the highest at $29,097, significantly exceeding that of cases with major or full liability (*p < 0.001*). Additionally, the number of catastrophic compensation cases was also highest in the main liability category, comprising 34.42% (159/462) of such cases. Although the cases where hospitals bore major or full liability represented only 13.73% (1,144/8,331) of the total, the high compensation rate was the highest at 12.33%.

### Distribution of surgical departments in medical malpractice cases

**Figure 5:**
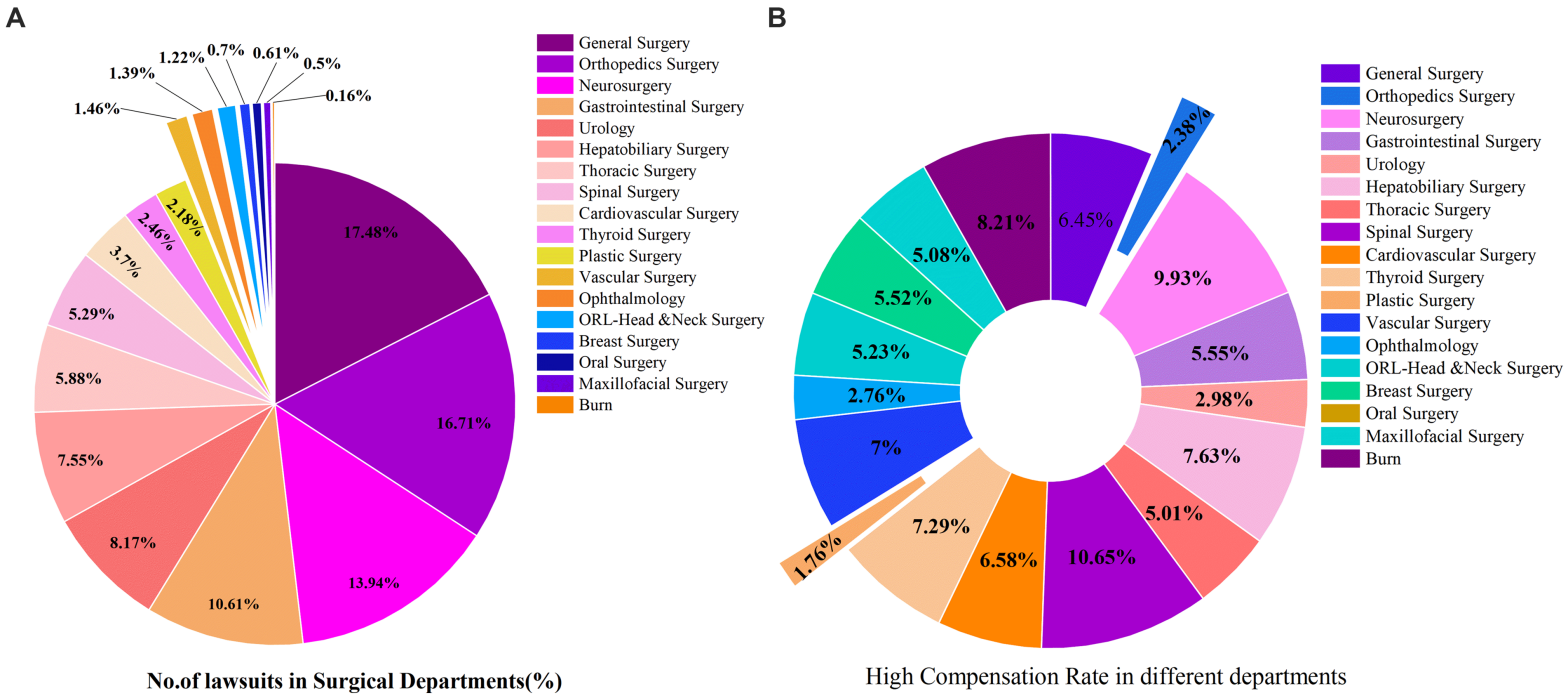
Relevant surgical departments. **(A)** Incidence rate of surgical medical disputes. **(B)** Risk of high compensation in surgical departments.

This study identified a total of 18 surgical departments involved in medical malpractice cases (Supplementary Table S1). Most cases occurred in general surgery, representing 17.48% (1,456/8,331) of the total. This was followed by orthopedic surgery, which accounted for 16.71% (1,392/8,331), and neurosurgery, with 13.94% (1,161/8,331) of the cases (Figure 5A). In terms of compensation, the top three surgical departments by median compensation amount were cardiovascular surgery ($28,160), neurosurgery ($27,003), and spinal surgery ($25,461). Notably, spinal surgery had the highest rate of high compensation claims, at 9.98%, followed by neurosurgery with a high compensation rate of 9.30% (Figure 5B).

### Multivariate logistic regression analysis of independent risk factors for catastrophic compensation

Based on correlation analysis, we initially identified several variables associated with catastrophic compensation in surgical medical malpractice cases. To further explore the risk factors, cases with compensation amounts exceeding $100,000 were defined as Y = 1, while cases with compensation amounts not exceeding $100,000 were defined as Y = 0. This classification served as the dependent variable in this study. We incorporated the influencing factors of surgical medical malpractice litigation as independent variables into a multivariate logistic regression analysis to identify potential risk factors contributing to catastrophic compensation in surgical medical malpractice disputes.

The multivariate logistic regression analysis revealed that catastrophic compensation is associated with hospital level, geographic region, degree of hospital responsibility, injury outcomes, and surgical department (Table 3). Specifically, primary and secondary hospitals are protective factors against catastrophic compensation compared to tertiary hospitals. Independent risk factors for catastrophic compensation in surgical medical malpractice disputes include hospitals located in the eastern region, secondary liability, main liability, major or full liability, severe disability, and surgical departments such as neurosurgery, thoracic surgery, general surgery, hepatobiliary surgery, gastrointestinal surgery, cardiovascular surgery, vascular surgery, and spinal surgery. These associations are statistically significant.

## Discussion

### Current status of surgical medical malpractice cases

This study includes 8,331 surgical medical malpractice cases. Between 2008 and 2019, there was a general upward trend in the number of such cases. However, from 2020, there has been a significant decline in case numbers. This reduction may be attributed to the COVID-19 pandemic that began in early 2020. Due to proactive healthcare policies and positive media coverage by the Chinese government, the doctor-patient relationship in China has notably improved (34, 35). Additionally, policies such as traffic lock downs, home isolation, and stringent protective measures during medical consultations have greatly reduced both the movement of people and the number of medical consultations (36), leading to a decrease in accidental injuries (37, 38) and surgical procedures (39). Thus, the reduction in surgical medical malpractice cases starting in 2020 may be related to the pandemic. Unfortunately, there has been no current research on the impact of the pandemic on medical disputes. However, with the global pandemic ending in 2023, this trend may change. Therefore, medical institutions and healthcare professionals should be prepared and enhance hospital quality control, as the number of surgical medical malpractice cases may rebound.

In this study, the total compensation amount for surgical medical malpractice cases was $269,163,545, representing a substantial economic burden on the development of China’s healthcare sector. The criteria for catastrophic compensation differ across countries. In the United States, catastrophic compensation is typically defined as exceeding $1 million (40). Spain defines it as exceeding $28,571 (41). Given China’s economic conditions and previous research, we applied a threshold of compensation exceeding $100,000 (33). In this study, there were 462 cases (5.55%) that met the catastrophic compensation criteria, totaling $71,392,976, which represents 26.52% of the overall compensation amount. This underscores the significant economic strain that catastrophic compensation cases place on public health.

### Eastern China as a risk factor for catastrophic compensation

Compared with other regions in China, Eastern China exhibits higher rates of surgical medical malpractice incidents and greater compensation amounts. This finding aligns with the results of Wang et al. (42). The disparity may be attributed to differences in population distribution and economic development among the regions. Eastern China not only has the largest population, but also significantly higher *per capita* disposable income compared to other regions. The population of the eastern region accounts for 39.39% of the total population in China. It has the highest *per capita* income, and the proportion of medical malpractice cases in surgical healthcare reaches 47.19%. Consequently, with its abundant labor and capital, Eastern China often benefits from superior medical resources (43).

Our study indicates that Eastern China is an independent risk factor for catastrophic compensation. This may be due to the higher economic development in the region, leading to elevated expectations for hospital services and medical quality. It may also because that the Eastern region of China, with its developed economy and dense population, has a higher number of tertiary hospitals and advanced medical standards, leading to the admission of more complex and critical patients. As a result, there are more unfavorable treatment outcomes. In contrast, the central, western, and northeastern regions, due to economic and population factors, mostly have private and smaller hospitals that treat fewer patients with less severe illnesses, which are less likely to result in medical disputes. This difference could further explain the higher incidence of litigation in the Eastern region. This highlights the need for medical institutions and healthcare professionals in Eastern China to focus on advancing medical technology and service quality. Specifically, surgical techniques and equipment should be updated regularly to meet patients’ growing demands, improve patient satisfaction, and reduce the incidence of medical disputes.

### Proportional liability of hospitals and catastrophic compensation

The findings of this study indicate a significant correlation between catastrophic compensation and the proportional liability of hospitals. Specifically, secondary liability (OR = 2.457, 95% CI 1.633–3.696), main liability (OR = 9.353, 95% CI 6.195–14.121), and major or full liability (OR = 10.878, 95% CI 7.152–16.546) are all identified as risk factors for catastrophic compensation, with the association becoming stronger as the proportion of liability increases. This suggests that the greater the proportion of liability shouldered by the hospital, the greater its contribution to the harm outcomes in surgical disputes, thereby increasing the risk of catastrophic compensation amounts. Consequently, the liability of the hospital is a key determinant in the amount of compensation awarded by courts. A similar conclusion was drawn by Shi et al. in their study of obstetric and gynecological medical liability disputes (33).

#### Tertiary hospitals and high compensation

The study results reveal that 70.60% of hospitals frequently named as defendants are tertiary hospitals. Furthermore, the high compensation rate for tertiary hospitals is 6.49%, which is 2–3 times greater than that of hospitals at other levels. Tertiary hospitals in China offer the highest level of medical care. According to China’s tiered healthcare system, patients with less severe conditions are treated at private, primary, or secondary hospitals, while those with severe or critical conditions are referred to tertiary hospitals. Even patients initially treated at lower-level hospitals may be transferred to tertiary hospitals as their conditions deteriorate. Due to the challenging journey and rapid disease progression, diagnostic delays are common upon arrival at tertiary hospitals. Consequently, many injury outcomes occur in tertiary hospitals. Moreover, the advanced medical capabilities of tertiary hospitals often lead patients and their families to place high expectations on them. As a result, even if the injury outcomes are not directly caused by the tertiary hospitals, these facilities frequently become targets of blame, contributing to a higher incidence of medical disputes and catastrophic compensation claims. This finding aligns with previous research (44, 45).

This highlights that tertiary hospitals, especially those frequently involved as defendants, should derive lessons from past medical disputes. When dealing with critically ill patients, immediate implementation of stress management strategies is essential. This includes establishing green channels, enhancing medical quality management, and strengthening risk identification and early warning mechanisms. Particularly when a patient’s condition is severe and may lead to death or an unsatisfactory treatment outcome, maximum communication should be conducted. Previous studies have addressed risk management and prediction related to medical disputes, reporting evidence-based strategies for managing medical risks, which suggest that medical institutions can implement preventive measures through early detection and intervention (5). Furthermore, it is necessary for hospital risk management departments to develop medical dispute risk prediction models to identify high-risk scenarios in surgical disputes, increase medical staff’s awareness of medical laws, and improve preventive measures against medical disputes (46).

#### Catastrophic compensation due to severe injury outcomes

In this study, cases resulting in patient death accounted for 56.99% of the total. Analysis reveals that the total compensation for death amounts to $153,851,405, exceeding half of the total compensation, which indicate that death is the most prevalent and costly outcome in surgical medical malpractice cases. However, a detailed analysis shows that the median compensation for cases resulting in severe and moderate disability exceeds that for death (*p* < 0.05). Furthermore, the study identifies severe disability as an independent risk factor for catastrophic compensation. Our findings show that the median compensation for severe disability is nearly twice that for death, consistent with previous research (45). Therefore, considering the long-term costs and subsequent loss of social productivity associated with patient injuries is a crucial factor in determining compensation. Given the burden of complex injury outcomes, healthcare professionals must understand the characteristics of cases leading to severe disability from past surgical litigations. Effective pre-treatment or preoperative communication is essential to mitigate the significant psychological impact on patients and their families, thereby reducing the incidence of surgical medical disputes.

### High-risk surgical departments for catastrophic compensation

This study examined surgical departments in the 18 specialty areas involved in medical malpractice cases, revealing a strong association between catastrophic compensation and departments such as spinal surgery, neurosurgery, general surgery, hepatobiliary surgery, and gastrointestinal surgery. Spinal surgical departments were found to have the highest rate of high compensation (9.98%), followed closely by neurosurgery (9.30%). Both spinal and neurosurgical departments deal with conditions involving the nervous system. Thus, conditions arising from either injury or postoperative complications can result in extremely severe outcomes, such as visual impairment, quadriplegia, or vegetative state. These profound consequences not only impose significant physical and psychological burdens on patients but also result in long-term economic costs for their families. Research by Jackson et al. has indicated that severe postoperative complications and paralysis are associated with significantly higher compensation amounts (47). Additionally, previous studies have shown that the risk of catastrophic compensation is notably higher in spinal surgery (48). This may be attributed to the fact that patients in neurosurgery often present with severe injuries, frequently accompanied by unconsciousness or impaired consciousness following trauma. Consequently, family members may be more psychologically prepared for potential postoperative complications, thereby making them more accepting of adverse outcomes (49). However, the high incidence and complexity of neurosurgical conditions also render this specialty prone to significant medical malpractice claims and compensations. In recent years, advancements in neurosurgical techniques have led to remarkable outcomes, particularly in the treatment of benign tumors, with minimally invasive techniques flourishing (50, 51). However, long-term and permanent postoperative complications (such as persistent ptosis and sensory deficits), particularly recurrence of tumor disease due to incomplete tumor resection, are more likely to result in severe adverse outcomes for patients, thereby increasing the occurrence of surgical malpractice disputes and catastrophic compensation.

Additionally, general surgery, hepatobiliary surgery, and gastrointestinal surgery are strongly associated with the risk of catastrophic compensation. Notably, these departments are closely interconnected, dealing with a wide range of diseases with similar characteristics. In some hospitals, patients are not distinctly categorized into separate departments but are instead managed under general surgery. Most of these conditions involve abdominal cavity organ disorders, which often progress rapidly and can be life-threatening. Given the complexity and proximity of adjacent organs within the abdominal cavity, the rates of misdiagnosis, missed diagnosis, and treatment delays are relatively high, leading to a higher incidence of medical disputes in these specialties (52, 53). Moreover, the necessity for invasive procedures and emergency surgeries in these departments contributes to a high incidence of postoperative complications, resulting in severe outcomes (54–56). Consequently, general surgery, hepatobiliary surgery, and gastrointestinal surgery are strongly correlated with substantial compensation, consistent with previous research findings (52, 53, 57).

Given the impact of various surgical departments on medical dispute compensation, healthcare institutions should enhance risk prevention education across all surgical departments, particularly those strongly associated with catastrophic compensation. This underscores the need for a proactive approach to dispute prevention within these departments. Both administrators and healthcare professionals should be well-informed about the specific disease characteristics of their departments and engage in regular legal training to increase legal awareness. Healthcare providers should adhere strictly to clinical guidelines during diagnosis and treatment, implement effective risk management strategies, and take measures to protect themselves from medical disputes. Such practices are crucial for improving surgical and nursing care quality (58).

## Limitations

This study conducted a retrospective analysis based on surgical medical malpractice cases retrieved from a database, which limited our ability to obtain comprehensive information about the origins of these disputes, such as the specific causes of the disputes. Consequently, we were unable to analyze the root causes of surgical medical malpractice. Additionally, our analysis was based solely on data from a single online database, which may not encompass all surgical medical malpractice cases occurring in China during the study period. Furthermore, this study only included cases that ended in litigation and did not analyze disputes resolved through settlement or mediation. Future research is encouraged to update this study and validate the robustness of the results.

## Conclusion

This study analyzes the characteristics and overall trends of surgical medical malpractice cases from multiple perspectives and identifies independent risk factors associated with catastrophic compensation. The insights gained are valuable for medical institutions and healthcare professionals in developing strategies to prevent and reduce surgical medical disputes, ultimately aiming to enhance the quality of medical and nursing care provided to patients.

## Data Availability

The datasets presented in this study can be found in online repositories. The names of the repository/repositories and accession number(s) can be found at: Jufaanli.

## Footnotes

^1^https://www.jufaanli.com/ (accessed 26 July 2024).

^2^www.stats.gov.cn (accessed 26 July 2024).
