# Supplementary material for: Improving surgical quality of care: learning from 8,331 surgical medical malpractice cases
Source: Front Med (Lausanne). 2024 Dec 10;11:1486451. doi: 10.3389/fmed.2024.1486451 (PMC11667895; doi:10.3389/fmed.2024.1486451)
Supplement: Supplementary file 1 [file Table_1.DOCX]

|  | | | | |
| --- | --- | --- | --- | --- |
| Supplementary Table 1**.** Relevant Surgical Departments and Compensation | | | | |
|  | | | | |
| **Departments in dispute** | **Total**  **n (%)** | **Without High Compensation**  **n (%)** | **With High Compensation n (%)** | **Median(IQR) Indemnity Compensation,**  **$** |
| Dept of Cardiovascular Surgery | 308(3.7) | 289(93.83) | 19(6.17) | 28160(13560-50562) |
| Dept of Neurosurgery | 1161(13.94) | 1053(90.70) | 108(9.30) | 27003(10701-52682) |
| Dept of Spinal Surgery | 441(5.29) | 397(90.02) | 44(9.98) | 25461(11023-51064) |
| Dept of Burn | 13（0.16） | 12(92.31) | 1(7.69) | 25176(10931-36111) |
| Dept of Gastrointestinal Surgery | 884(10.61) | 838(94.80) | 46(5.20) | 23415(10230-44279) |
| Dept of Thoracic Surgery | 490(5.88) | 467(95.31) | 23(4.69) | **23363(11849-44044)** |
| Dept of Thyroid Surgery | 205(2.46) | 191(93.17) | 14(6.83) | 17788(5994-36180) |
| Dept of Hepatobiliary Surgery | 629(7.55) | 584(92.85) | 45(7.15) | 22115(9288-45519) |
| Dept of General Surgery | 1456(17.48) | 1368(93.96) | 88(6.04) | 21696(8422-45528) |
| Dept of Breast Surgery | 58(0.70) | 55(94.83) | 3(5.17) | 20521(7941-39532) |
| Dept of Vascular Surgery | 122(1.46) | 114(93.44) | 8(6.56) | 18098(7564-43054) |
| Dept of Urology | 681(8.17) | 662(97.21) | 19(2.79) | 17785(8418-33823) |
| Dept of Maxillofacial Surgery | 42(0.50) | 40(95.24) | 2(4.76) | 15450(3513-33729) |
| Dept of ORL-Head &Neck Surgery | 102(1.22) | 97(95.10) | 5(4.90) | 14349(4275-36294) |
| Dept of Orthopedics Surgery | 1392(16.71) | 1361(97.77) | 31(2.23) | 12603(5233-26213) |
| Dept of Ophthalmology | 116(1.39) | 113(97.41) | 3(2.59) | 11382(4974-25419) |
| Dept of Plastic Surgery | 182(2.18) | 179(98.35) | 3(1.65) | 6985(3142-20314) |
| Dept of Oral Surgery | 51(0.61) | 51(100) | 0(0) | 2970(815-12902) |
| ***Fisher-Freeman-Halton Exact Test=*** ***110.385 p<0.001***  Dept, departments of surgery | | | | |
