# Supplementary material for: Improving surgical quality of care: learning from 8,331 surgical medical malpractice cases
Source: Front Med (Lausanne). 2024 Dec 10;11:1486451. doi: 10.3389/fmed.2024.1486451 (PMC11667895; doi:10.3389/fmed.2024.1486451)
Supplement: Supplementary file 2 [file Table_2.docx]

| **Author** | **Country** | **Year** | **Department** | **No. cases(n)** |
| --- | --- | --- | --- | --- |
| Gerstl J V E | America | 2024 | Neurosurgery | 1550 |
| Fadel M A | America | 2023 | Otolaryngology | 28 |
| Chao J C | America | 2023 | Thyroid Surgery | 68 |
| Larkin C J | America | 2020 | Neurosurgery | Not mentioned |
| Remington A C | America | 2024 | Plastic Surgery | 2674 |
| Green M A | America | 2022 | Maxillofacial Surgery | 1445 |
| Panuganti P L | America | 2020 | Gastrointestinal Surgery | 240 |
| Tang O Y | America | 2020 | Neurosurgery | 113 |
| Sauder N | America | 2023 | Orthopedics Surgery | 164 |
| Megalla M | America | 2023 | Orthopedic Surgery | 35 |
| Hu D | China | 2023 | Spine Surgery | 186 |
| Barré L | America | 2022 | Several departments | 460 |

Supplementary Table 2**.** Previous research on surgical medical disputes
